# USP7 is a novel Deubiquitinase sustaining PLK1 protein stability and regulating chromosome alignment in mitosis

**DOI:** 10.1186/s13046-019-1457-8

**Published:** 2019-11-15

**Authors:** Yuchong Peng, Youhong Liu, Yingxue Gao, Bowen Yuan, Xuli Qi, Yuxin Fu, Qianling Zhu, Tuoyu Cao, Songwei Zhang, Linglong Yin, Xiong Li

**Affiliations:** 1 Center for Molecular Medicine, Xiangya Hospital, Central South University, Changsha, China; 2 Hunan Key Laboratory of Molecular Radiation Oncology, Xiangya Hospital, Central South University, Changsha, China; 3 Department of Pathology, The Third Xiangya Hospital, Central South University, Changsha, China; 4 School of Clinical Pharmacy, Guangdong Pharmacology University, Guangzhou, China; 5 The First Affiliated Hospital, Guangdong Pharmacology University, 19 Nonglinxia Road, Yuexiu District, Guangzhou, Guangdong China

**Keywords:** USP7, PLK1, Chromosome misalignment, Cell cycle arrest, Apoptosis

## Abstract

**Background:**

The deubiquitinase USP7 has been identified as an oncogene with key roles in tumorigenesis and therapeutic resistance for a series of cancer types. Recently small molecular inhibitors have been developed to target USP7. However, the anticancer mechanism of USP7 inhibitors is still elusive.

**Methods:**

Cell viability or clonogenicity was tested by violet crystal assay. Cell apoptosis or cell cycle was analyzed by flow cytometry, and chromosome misalignment was observed by a fluorescent microscopy. The protein interaction of PLK1 and USP7 was detected by tandem affinity purification and high throughput proteomics, and further confirmed by co-immunoprecipitation, GST pull-down and protein co-localization. The correlation between USP7 level of tumor tissues and taxane-resistance was evaluated.

**Results:**

Pharmacological USP7 inhibition by P5091 retarded cell proliferation and induced cell apoptosis. Further studies showed that P5091 induced cell cycle arrest at G2/M phase, and particularly induced chromosome misalignment, indicating the key roles of USP7 in mitosis. USP7 protein was detected in the PLK1-interacted protein complex. USP7 interacts with PLK1 protein through its PBD domain by catalytic activity. USP7 as a deubiquitinase sustained PLK1 protein stability via the C223 site, and inversely, USP7 inhibition by P5091 promoted the protein degradation of PLK1 through the ubiquitination-proteasome pathway. By overexpressing PLK1, USP7 that had been depleted by RNAi ceased to induce chromosome misalignment in mitosis and again supported cell proliferation and cell survival. Both USP7 and PLK1 were overexpressed in taxane-resistant cancer cells, and negatively correlated with the MP scores in tumor tissues. Either USP7 or PLK1 knockdown by RNAi significantly sensitized taxane-resistant cells to taxane cell killing.

**Conclusion:**

This is the first report that PLK1 is a novel substrate of USP7 deubiquitinase, and that USP7 sustained the protein stability of PLK1. USP7 inhibition induces cell apoptosis and cell cycle G2/M arrest, and overcomes taxane resistance by inducing the protein degradation of PLK1, resulting in chromosome misalignment in mitosis.

## Background

Protein stability is critical for normal cellular homeostasis. In addition to the autophagy-lysosome system, the ubiquitin-proteasome system (UPS) takes up approximately 80 to 90% of intracellular protein degradation [1]. In UPS-induced protein degradation, ubiquitin binds to target proteins and catalyzes them by a hierarchical cascade comprising E1, E2 and E3 ubiquitin ligases [2]. Inversely, the ubiquitination is removed from the labeled proteins or from polyubiquitin chains by deubiquitinating enzymes (or deubiquitinases, DUBs). DUBs are critical in cellular growth, survival and homeostasis, and are responsible for the turnover, localization and activity of their substrate proteins. Aberrant DUB activity results in a series of diseases, including cancer [3, 4].

Ubiquitin-specific proteases (USPs) are the largest DUB in all subfamilies, of which USP7 is the most prominent and well characterized member [5]. USP7 was originally identified as a binding partner for the herpes simplex virus (HSV) infected cell protein and named herpes-associated ubiquitin-specific protease (HAUSP) [6]. USP7 plays an important role in the cancer-related p53-MDM2 network [7–9]. USP7 specifically dequbiquitinates and stabilizes both p53 and MDM2 to various degrees, and USP7 inhibition is expected to inactivate MDM2 and activate p53, thereby leading to cell cycle arrest or apoptosis in cancer cells with functional p53 signaling [10]. In addition, USP7 promotes cell proliferation by stabilizing Ki-67 protein [11]. USP7 is also involved in other cancer-associated mechanisms such as DNA damage and repair [12], epigenetic regulation [13], human terminal erythoid differentiation [14] and immune responses by regulating other cancer-related targets such as N-Myc [15], FOXO, PTEN and Claspin [5, 16]. USP7 is the first USP recognized as one of the cancer therapeutic DUB targets due to its important roles in tumorigenesis, cancer metastasis and HIV progression [17]. Several small molecular inhibitors of USP7 have been developed and are being tested in clinical trials [18]. The available data suggest that USP7 inhibitors induce cell cycle arrest and apoptosis in cancer cells through the p53 pathway, and sensitize cancer cells to PARP inhibitor-induced cell death [18]. P5091, a selective USP7 inhibitor, induces cell apoptosis by blocking the Wnt-β-catenin pathway [19]. Additionally, P5091 has an important role in anticancer immunity in the tumor microenvironment by inhibiting FOXP3 expression [20].

In addition to its roles in carcinogenesis, USP7 plays a critical role in therapeutic resistance. USP7-mediated MDC1 stabilization promotes cervical cancer cell survival and conferred cellular resistance to genotoxic insults [21]. USP7 knockdown overcomes Bortezomib resistance by suppressing the NF-kB signaling pathway in multiple myeloma [22]. USP7 inhibitors show great efficacy for inhibiting myeloma cell growth and overcoming NEK2-induced and acquired drug resistance in xenograft myeloma mouse models [23]. USP7 inhibition sensitizes p53-defective, chemotherapy-resistant chronic lymphoblastic leukemia (CLL) cells to clinically achievable doses of homologous recombination repair (HRR)-inducing chemotherapeutic agents in vitro and in vivo in a murine xenograft model [24]. Mitotic aberrance induces cell cycle arrest and apoptosis. A large number of molecules have been identified to be involved in the regulation of mitosis. The roles of USP7 in the regulation of mitosis have not been reported.

PLK1 is a master mitotic regulator controlling a wide variety of processes during G2/M cell cycle progression, such as centrosome maturation, entry into mitosis, chromosome segregation and cytokinesis. In prophase, PLK1 shuttles to the kinetochores, helping condensed chromosomes to align in the metaphase plate. At the onset of anaphase, PLK1 localizes at the spindle mid-zone and later to the cytokinetic bridge in order to coordinate cytokinesis and cell abscission. PLK1 has strong clinical relevance. It is overexpressed in many cancer types, and the degree of intratumoral overexpression correlates with poor patient prognosis [25, 26]. PLK1 has been considered a bona fide cancer target, and PLK1 inhibition results in aberrance of mitotic progression. PLK1 inhibition prevents the formation of a bipolar spindle, thus preventing the proper alignment of chromosomes in the metaphase plate. PLK1 inhibitors have been developed to experimentally treat different cancer types. Furthermore, PLK1 hyper-activation contributes to cancer therapeutic resistance to chemotherapy and radiotherapy [27]. Inversely, PLK1 depletion re-sensitizes cancer cells to therapy, overcoming therapeutic resistance.

In the present study, we for the first time found that USP7 inhibition induced chromosome misalignment in mitosis, thereby inducing G2/M cell cycle arrest and apoptosis. The possible mechanism is that USP7 protein physically interacts with PLK1 protein, and USP7 depletion induces the protein degradation of PLK1, thereby resulting in mitotic aberrance. Overexpressing PLK1 after USP7 knockdown by RNAi blocked the ability to induce chromosome misalignment in mitosis, while USP7 inhibition by P5091 retarded cell proliferation and cell survival and overcame taxane resistance. These data suggest that USP7 inhibitors induce mitotic aberrance by regulating PLK1 function, and thus show great potential for cancer therapy.

## Methods

### Cell culture and viral infection

DU145, VCaP and HEK293T cells were obtained from American Type Culture Collection (Manassas, VA, USA). Nasopharyngeal cancer (NPC) cell line CNE2 was purchased from the Cancer Research Institute of Central South University (Changsha, Hunan, China). The paclitaxel-resistant NPC cell subline CNE2-TR and docetaxel-resistant cell subline DU145-DR were established by intermittently exposing the parental cells to gradually increasing concentrations of paclitaxel or docetaxel. Cells were maintained in DMEM or 1640 medium supplemented with 10% fetal bovine serum (FBS), 1% penicillin, and streptomycin. Stable cell lines expressing USP7 shRNA were generated by transfecting DU145 or VCaP cells with PLKO.1/PLKO.1-USP7 shRNA plasmids, and were selected by 1 μg/ml puromycin. All cells were cultured in a humidified incubator at 37 °C and 5% CO_2_.

### Plasmids and siRNA transfection

pGEX-4 T-1-PLK1, pCMV-USP7-Flag, pCMV-PLK1-Flag and pCMV-PLK1 plasmids were constructed by generating the cDNA fragments of PLK1 and USP7 by PCR, and then subcloning them into the described vectors. To construct the USP7 and PLK1 mutants, we generated the cDNA fragments of wild-type or mutated PLK1 and USP7 by PCR, and then subcloned them into the above vectors. pLKO.1-USP7 shRNA plasmids were generated by subcloning USP7 shRNA into pLKO.1 lentiviral vector. (USP7 shRNA Sense: 5′-TGTATCTATTGACTGCCCTTT-3′).

siRNA transfection was performed using a Mirus transfection kit according to the manufacturer’s instructions (Mirus, Madison, WI, USA). The sequences of USP7 siRNA (5′-3′) are as follows: USP7#1 (UGUAUCUAUUGACUGCCCU), USP7#2 (CGUGGUGUCAAGGUGUACU) and PLK1 (CACCAUAUGAAUUGUACAG).

### RNA extraction and real-time quantitative PCR

Total RNA was isolated using the RNAiso Plus method following the manufacturer’s protocol (Takara Bio Inc., Beijing, China). The concentration and quality of dissolved RNA samples was determined by a spectrophotometer before the conversion to cDNA. Quantitative real-time PCR was then performed using a real-time PCR detection system (Thermo Fisher Scientific 7500) according to the manufacturer’s instructions (Waltham, MA, USA).

### Antibodies and reagents

Antibodies and reagents used in the study and their sources are listed as follows: anti-USP7, anti-PLK1, anti-Ubiquitin, anti-α-tubulin, mouse IgG were purchased from Cell Signal Transduction (CST, Danvers, MA, USA). Anti-β-actin antibody was purchased from Santa Cruz Biotechnology (Dallas, UT, USA), anti-Flag was purchased from Sigma Aldrich (St. Louis, MO, USA) and rabbit IgG was purchased from Protein-tech (Wuhan, China). Anti-USP7 antibody for immunohistochemistry was purchased from ABclonal Technology (Upper Heyford, UK). The USP7 inhibitor P5091 was purchased from MedChemExpress (MCE, Shanghai, China), CHX was purchased from CST, and MG132 was purchased from Selleck Chemicals (Houston, Texas, USA).

### Immunoblotting and immunoprecipitation

Cells were lysed and protein was extracted using RIPA buffer supplemented with protease inhibitor cocktails (Selleck Chemicals). Protein concentration was measured with a Bradford assay kit (Thermo Fisher Scientific). An equal amount of protein was loaded and separated by SDS-polyacrylamide gel electrophoresis (SDS-PAGE) with β-actin as the loading control.

Extracts for immunoprecipitation were prepared using RIPA buffer supplemented with protease inhibitor cocktails (Selleck Chemicals). The extracts were then incubated with the indicated antibodies at 4 °C for 12 h on a rotator, followed by incubation with proteinA/G-magnetic beads (MCE) at 4 °C for 1 h on a rotator. After incubation, beads were washed four times in immunoprecipitation buffer and boiled in 1 × loading buffer. Protein samples were analyzed by SDS-PAGE.

### GST pull-down assay

pGEX-4 T-1-PLK1 was constructed using the GST gene fusion system according to the manufacturer’s instructions. To produce glutathione S-transferase (GST) and GST-PLK1 fusion proteins, pGEX-4 T-1 and pGEX-4 T-1-PLK1 were individually transfected into BL21 complete cells. Protein expression was induced by ispropyl b-D-1-thiogalactopyranoside (IPTG). GST-PLK1 and GST proteins were purified by binding to glutathione-sepharose resins (Sangon Biotech, Shanghai, China). Resins were incubated with cell lysates from DU145 cells at 4 °C for 12 h on a rotator, and then were washed with the washing buffer four times. Resin-bound complexes were eluted by boiling, and subjected to western blotting.

### Cellular immunofluorescence

Cells grown on coverslips were fixed with 4% paraformaldehyde solution for 15 min at room temperature, permeabilized with 0.25% Triton X-100 for 20 min, and then blocked with 5% bovine serum albumin for 1 h. Cells were incubated with the primary antibodies as indicated at 4 °C for 12 h, and then incubated with the fluorescent-labeled secondary antibodies. The nuclei were stained with DAPI. Images were captured and analyzed with Zeiss fluorescence microscopy.

### Flow cytometry

For cell cycle analysis, cells were first fixed in 70% ethanol at 4 °C. DNA was stained with 0.02 mg /ml propidium iodide containing RNase (30 μg/ml) at 37 °C for 30 min. Cell cycle phase distribution was analyzed by flow cytometry (Millipore, Temecula, CA, USA).

Apoptosis was detected with an Annexin V-FITC kit. Briefly, cells were incubated with 5 μl Annexin V-FITC and 5 μl propidium iodide in 100 μl binding buffer for 15 min at room temperature, immediately followed by flow cytometry analysis.

### MTS cell viability assay

Cells were cultured in 96-well flat-bottomed microtiter plates (5000 per well), and treated with paclitaxel or docetaxel with or without P5091 for 24 h. Then 20 μl of MTS solution [3-(4,5-dimethylthiazol-2-yl)-5-(3-carboxymethoxyphenyl)-2-(4-sulfophenyl)-2Htetrazolium] (Promega, Madison, WI, USA) was added to each well, and cultured at 37 °C in 5% CO_2_ for 2 h. Absorbance at 490 nm was measured by spectrometer. Each treatment was performed in quintuplicate. The relative survival rate of the cancer cells was calculated according to the cell viability.

### Immunohistochemistry

Paraffin sections of breast tumor tissues were obtained from the Department of Pathology, Xiangya Hospital, Central South University. The slides were incubated with primary antibody against USP7. For negative control, isotype-matched antibodies were applied. USP7 positive signals were detected in both nuclear and cytoplasmic compartments. The IHC results were judged from 5 to 10 random fields (× 400) by two independent senior pathologists. The degree of staining was classified into four stages (− negative, + slight yellow, ++ brown, +++ dark brown or tan). The ratio of positive cells was divided into three stages (+ < 25%, ++ 25 to 49%, +++ ≥ 50%). The overall standard including staining degree and ratio of positive cells: +percent of positive cells × 1, ++ percent of positive cells × 2 and +++ percent of positive cells × 3. The overall results: + < 1, ++ 1 to 1.5, +++ > 1.5.

### Statistical analysis

All data were analyzed by Origin 8. Results are presented as mean ± SD. One-way ANOVA was used to analyze the statistical difference of multiple groups. *P < 0.05 and **P < 0.01. *P* < 0.05 was considered as statistically significant.

## Results

### USP7 depletion impaired cell colony formation and retarded cell proliferation by inducing G2/M cell cycle arrest and chromosome misalignment in mitosis

**Fig. 1:**
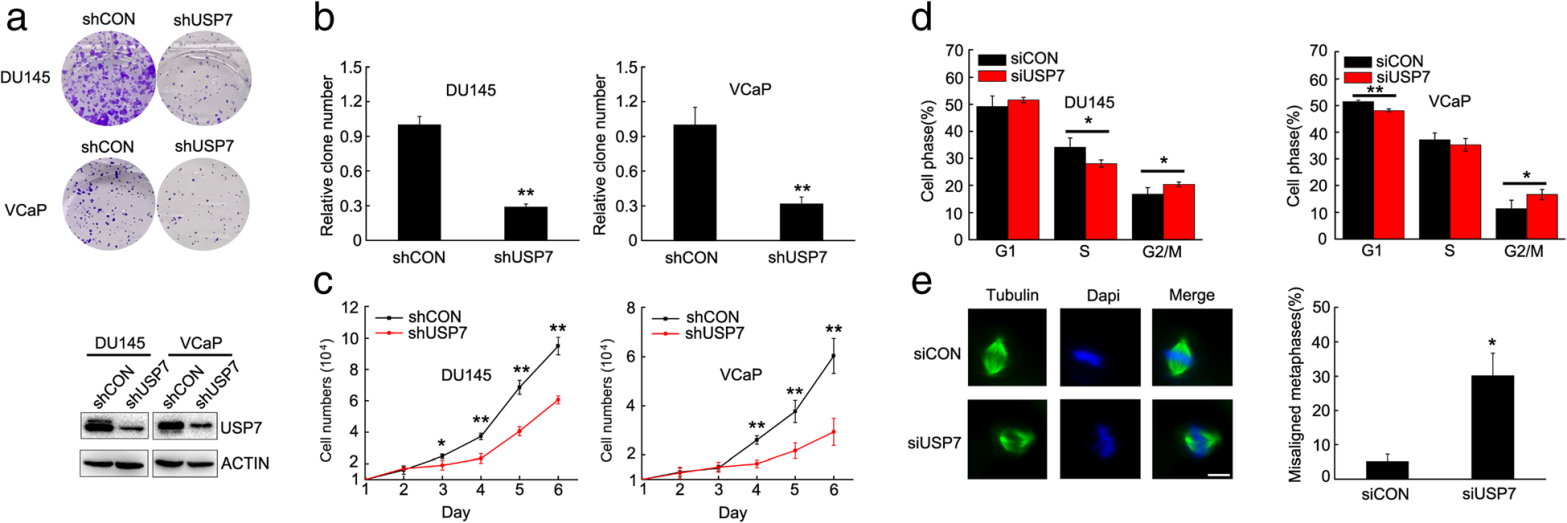
Depletion of USP7 retarded cell proliferation by inducing G2/M cell cycle arrest and chromosome misalignment in mitosis. **a** and **b** DU145 or VCaP cells stably expressing USP7 or control shRNA were generated. The same number of cells were seeded and the colonies formed were counted 14 days later. The knockdown efficiency of USP7 was tested by western blot. **c** The growth of DU145 or VCaP cells stably expressing USP7 or control shRNA were compared every day after cell seeding. Standard deviation bars were obtained from triplicate repeats. **d** The cell cycle distribution of DU145 or VCaP cells expressing USP7 or control siRNA were analyzed by flow cytometry. Standard deviation bars were obtained from three independent experiments. **e** In DU145 or VCaP cells expressing USP7 or control siRNA, spindles were stained with immunofluorescent α-tubulin antibody and nuclear DNA were stained with DAPI. The number of cells with misaligned chromosomes were quantified (scale bar, 10 μM)

USP7 has been identified as an oncogene, and plays a key role in tumorigenesis in several cancer types, including prostate cancer. We generated two cell lines, DU145 and VCaP with USP7 stable knockdown, by infecting cells with lentiviral vector expressing USP7 shRNA, and compared their ability to form cell colonies and proliferate. After USP7 knockdown the number of cell colonies significantly decreased (Fig. 1a and b) and cell proliferation was significantly retarded (Fig. 1c).

To further explore the mechanisms by which USP7 depletion impaired cell colony formation and retarded cell proliferation, we tested changes of cell cycle distribution when USP7 was knockdown with siRNA. Consistently in the two cell lines, USP7 depletion significantly increased the cell population at G2/M phase, indicating cell cycle arrest (Fig. 1d). We further monitored chromosome alignment in mitosis when USP7 was knockdown with siRNA. USP7 knockdown significantly elevated the percentage of cells with misaligned chromosome in mitosis (Fig. 1e). These results indicated that USP7 is a critical molecule with a key role in mitosis.

### PLK1 is a novel substrate of USP7, and USP7 sustains the protein stability of PLK1

**Fig. 2:**
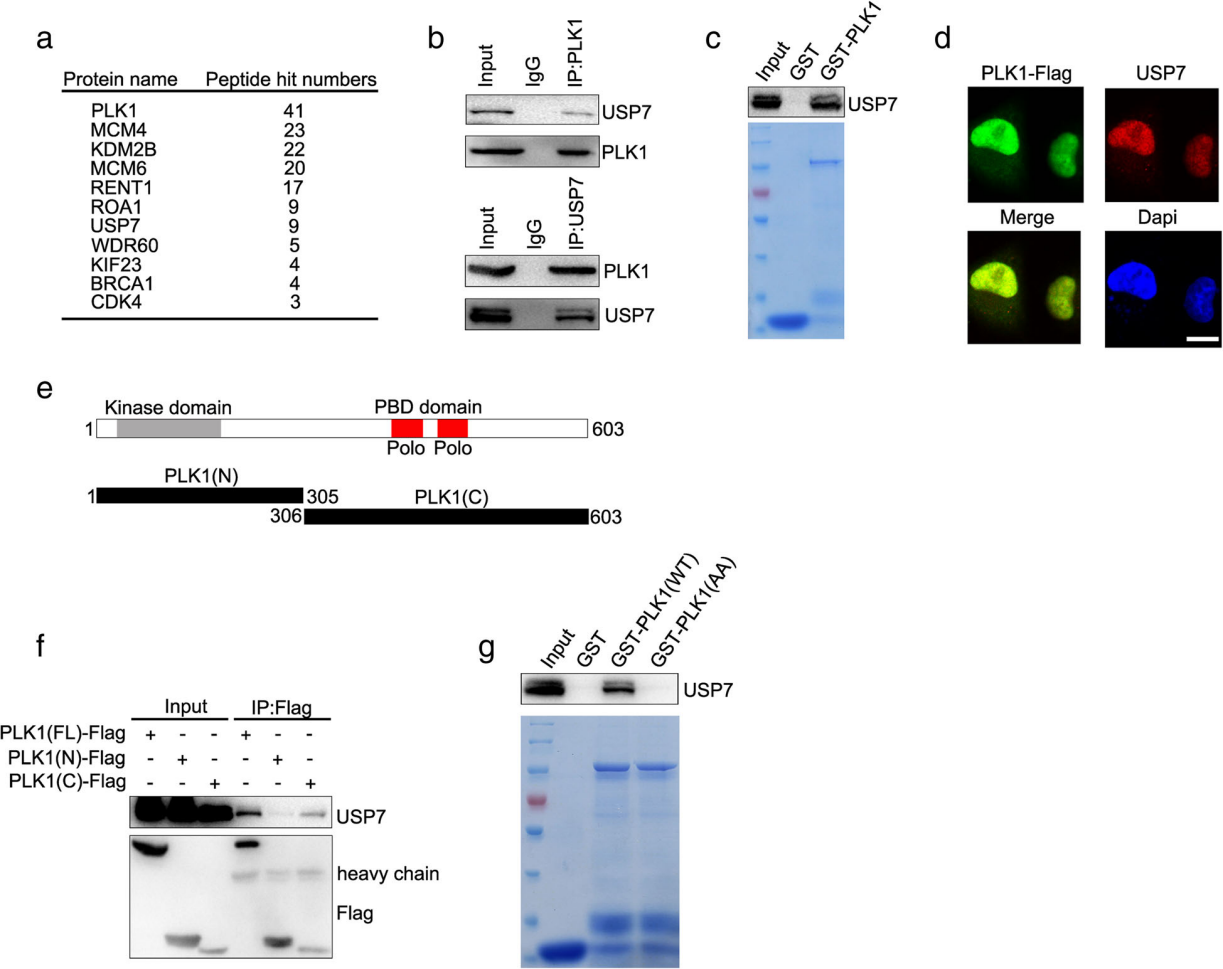
USP7 protein co-localizes and interacts with PLK1 protein. **a** Tandem affinity purification was performed using HEK293T cells stably expressing Flag-tagged PLK1. The major hits from mass spectrometry analysis are shown in the Table. **b** PLK1 protein interacts with USP7 protein. DU145 cells were lysed and immunoprecipitation and immunoblotting were performed using the indicated antibodies. **c** GST-pull-down assay of PLK1 using the indicated GST fusion proteins. USP7 protein was detected. **d** DU145 cells expressing PLK1-Flag were stained with anti-USP7 (red) and anti-Flag antibody (green). Cellular nuclei were labeled with DAPI. Scale bar, 10 μM. **e** Schematic diagram of PLK1 protein domains. **f** HEK293T cells were transfected with Flag-tagged plasmids expressing full length PLK1 or its truncated mutants as indicated. Cell lysates were immunoprecipitated with anti-Flag antibody, followed by immunoblotting with anti-USP7 antibody. **g** Purified recombinant proteins of GST, GST-PLK1(FL) and GST-PLK1(AA) were incubated with cell lysates in vitro as indicated, followed by immunoblotting with anti-USP7 antibody

In another ongoing research project, we screened PLK1-associated proteins by tandem affinity purification with an anti-Flag antibody, followed by high throughput proteomics by using a mass spectrometer. Beyond our expectation, USP7 was identified as a novel substrate of PLK1 at the top of the list of PLK1-interactive proteins (Fig. 2a). The protein interaction of USP7 and PLK1 was validated by co-immunoprecipitation. USP7 protein was detected when PLK1 was immunoprecipitated by PLK1 antibody, and inversely PLK1 was detected when USP7 was immunoprecipitated in DU145 cells (Fig. 2b). We further revealed that USP7 protein physically interacts with PLK1 by GST pull down assay (Fig. 2c). The protein interaction of USP7 and PLK1 was further validated in DU145 cells by observing their intercellular co-localization (Fig. 2d). The structure of PLK1 protein is divided into a N terminal fragment containing a kinase domain and a C terminal fragment containing a PBD domain (Fig. 2e). Co-immunoprecipitation validated USP7 interaction with the C terminal domain of PLK1 protein (Fig. 2f). H538A/K540A (AA) mutations that disrupt the protein-interacting capability of PBD [28] result in the loss of PLK1 and USP7 protein interaction (Fig. 2g). The polo box motifs of PLK1 protein are critical for its protein interaction with USP7.

### USP7 sustains the protein stability of PLK1 as a deubiquitinase

**Fig. 3:**
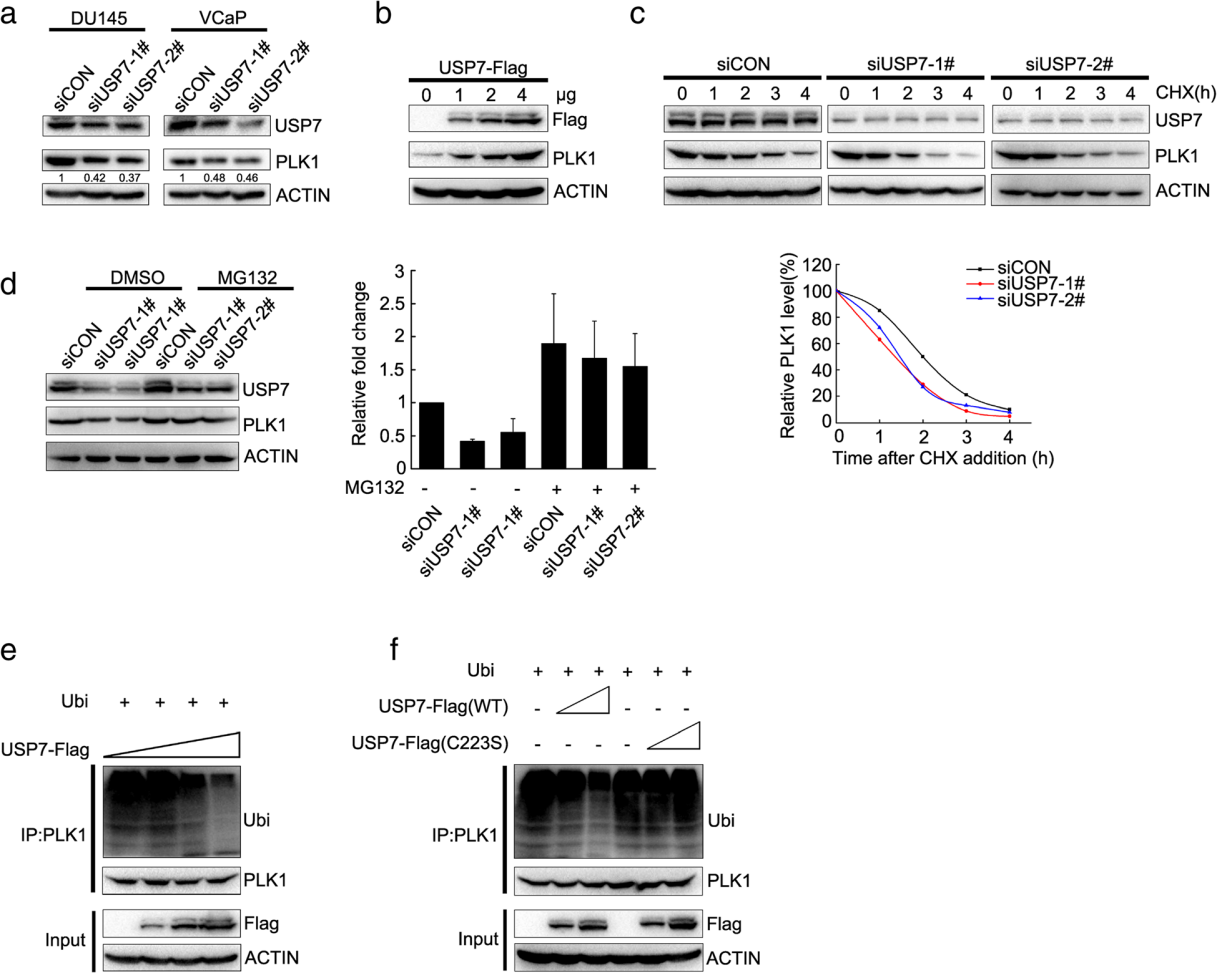
USP7 sustains the protein stability of PLK1 as a deubiquitinase. **a** DU145 and VCaP cells were transfected with scrambled or USP7 siRNAs. Protein levels of USP7, PLK1 and β-actin were assessed by immunoblotting. **b** DU145 cells were transfected with increasing amounts of pCMV-USP7-Flag as indicated. The PLK1 protein level was assessed by immunoblotting. **c** DU145 was transfected with scrambled or USP7 siRNAs. Cells were treated with 50 μg/mL cycloheximide (CHX) for the indicated times, and endogenous PLK1 protein levels were monitored over time by immunoblotting. PLK1 protein was quantified by greyscale analysis. **d** DU145 was transfected with scrambled or USP7 siRNAs. Cells were treated with MG132 for 8 h, and PLK1 or USP7 protein was detected by immunoblotting. PLK1 protein was quantified by greyscale analysis. Standard deviation represents three independent experiments (Additional file 4: Figure S4). **e** and **f** Impact of USP7 on PLK1 ubiquitylation in vitro. HEK293T cells were transfected with increasing amounts of pCMV-USP7-Flag or pCMV-USP7(C223S)-Flag together with pCDNA3.1-Ubi-HA and pCMV-PLK1, and then were treated with 25 μM MG132 for 8 h. Immunoprecipitation was performed with anti-PLK1 antibody, and subjected to western blot with an ubiquitin or PLK1 antibody

Since USP7 protein, as a deubiquitinase, physically interacts with PLK1 protein, we tested whether USP7 depletion influenced PLK1 protein stability. In both DU145 and VCaP cells, the depletion of USP7 significantly decreased PLK1 protein levels but no effect on PLK1 mRNA (Fig. 3a; Additional file 1: Figure S1A-B). Inversely, when USP7 protein levels were elevated by transient transfection, the protein level of PLK1 accordingly rose in a dose-dependent manner (Fig. 3b).

To explore the mechanism by which USP7 sustains PLK1 protein stability, we assessed the half-life of PLK1 protein when USP7 was depleted. The protein synthesis was inhibited by cycloheximide (CHX), and PLK1 protein was assessed at different time points. The knockdown of USP7 with siRNA promoted the protein degradation of PLK1 and shortened the half-life of PLK1 protein (Fig. 3c). The degradation of PLK1 protein was inhibited by MG132, an inhibitor of the ubiquitination-proteasome pathway, indicating that USP7 sustained PLK1 protein stability through the ubiquitination-proteasome pathway (Fig. 3d).

We further validated the results by co-transfecting the plasmids expressing HA-ubiquitin or PLK1 to HEK293T cells. PLK1 protein was immunoprecipitated, and the ubiquitinated protein was detected with ubiquitin antibody. The ubiquitin protein steadily decreased with the increase of USP7 (Fig. 3e). USP7 is a deubiquitinase, and C223 has been identified as the catalytic site for its deubiquitination function [29]. When C223 was mutated, the elevation of USP7 protein levels did not decrease PLK ubiquitinated protein levels (Fig. 3f). The results further validated that USP7 sustains PLK1 protein stability based on the deubiquitination catalytic activity of USP7.

### The USP7 inhibitor P5091 retarded cell proliferation and induced cell apoptosis and G2/M cell cycle arrest

**Fig. 4:**
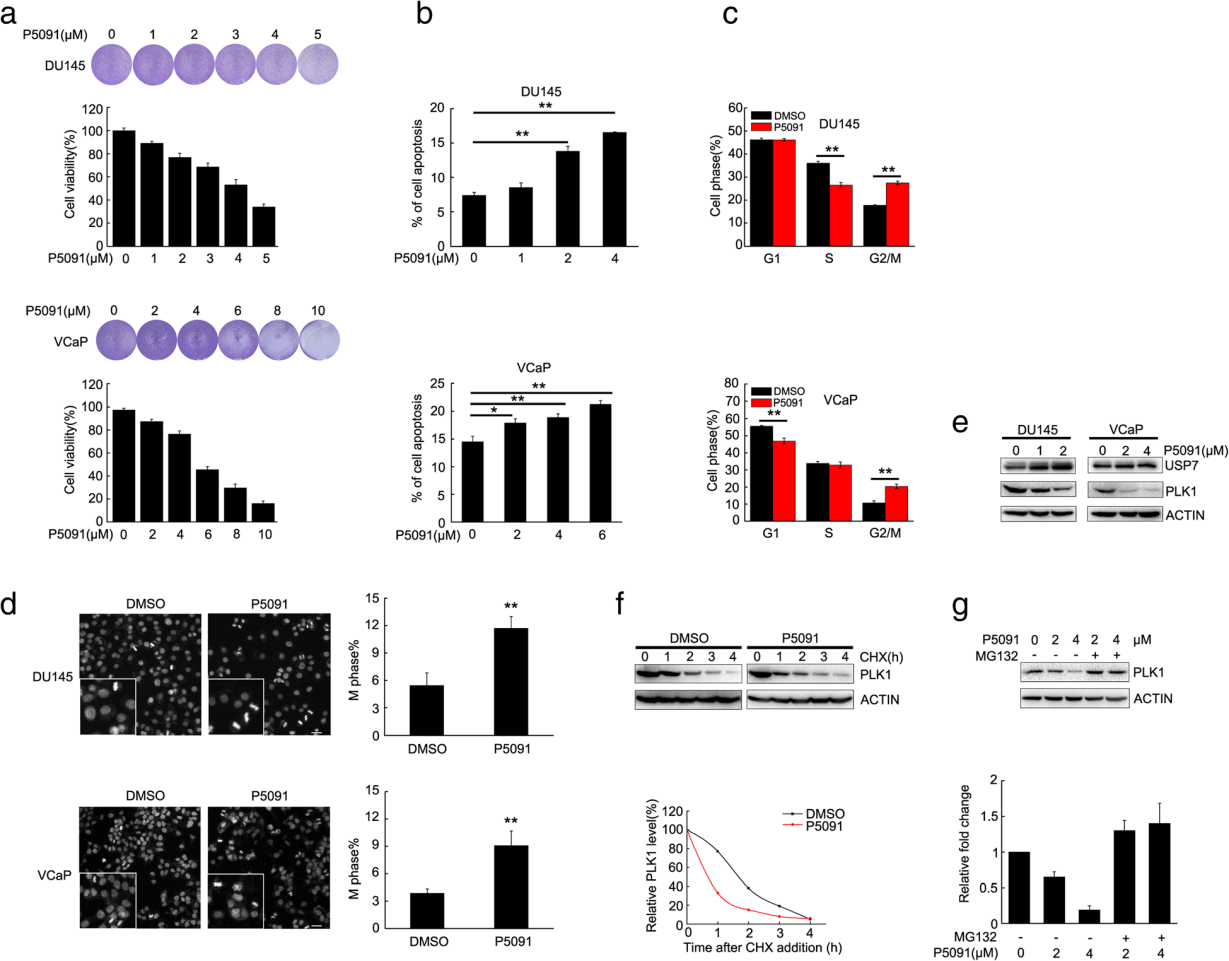
P5091 retarded cell proliferation and induced cell apoptosis and G2/M cell cycle arrest by degrading PLK1 protein. **a** DU145 and VCaP cells were treated with P5091 for 2 days. Cells were stained with crystal violet and then lysed with 1% SDS. Cell viability was assessed at OD570 nm by spectrometer. **b** DU145 and VCaP were treated with P5091 for 24 h. Cells were stained with FITC-Annexin V and propidium iodide (PI). The apoptotic cells were analyzed by flow cytometry. **c** DU145 or VCaP cells were treated with P5091 for 2 days. Cells were labeled with PI, and cell cycle status was analyzed by flow cytometry. **d** DU145 or VCaP cells were treated with P5091 for 2 days, and the DNA in nuclei was stained with DAPI. Scale bar, 20 μM. The percent of mitotic cells were assessed according to nuclei morphology. **e** DU145 and VCaP cells were treated with the USP7 inhibitor P5091 for 24 h, and the protein levels of USP7 and PLK1 were assessed by immunoblotting. **f** DU145 cells were treated with P5091 (1 μM) for 24 h, followed by continuous CHX (50 μg/mL) treatment for 1–4 h. PLK1 protein levels were analyzed by immunoblotting. PLK1 protein levels were calculated by grayscale analysis. **g** DU145 cells were treated with P5091 for 12 h, followed by treatment with 50 μM MG132 for 8 h. PLK1 protein was assessed by western blot. PLK1 protein levels were calculated by grayscale analysis

P5091, a selective USP7 inhibitor, has shown potential anticancer efficacy in pre-clinical trials [30, 31]. We tested the impact of P5091 on the viability of prostate cancer DU145 and VCaP cells. Consistently in these two cell lines, P5091 significantly decreased cell viability in a dose-dependent manner (Fig. 4a). We investigated whether P5091 decreased cell viability by inducing cell apoptosis and cell cycle arrest. P5091 induced cell apoptosis in a dose-dependent manner (Fig. 4b), and also induced G2/M cell cycle arrest (Fig. 4c). We further clarified whether P5091 induced G2/M cell cycle arrest by increasing mitotic cells. As shown in Fig. 4d, P5091 induced more mitotic cells based on nuclear morphology. P5091 induced the protein degradation of PLK1 in a dose-dependent manner (Fig. 4e) and shortened the half-life of PLK1 protein, when new protein synthesis was inhibited by CHX (Fig. 4f). PLK1 protein degradation could be inhibited by MG132 (Fig. 4g), validating that USP7 sustains PLK1 protein through the ubiquitination-proteasome pathway. The biological effects of the USP7 inhibitor were very similar to USP7 knockdown with RNAi.

### PLK1 rescued USP7 knockdown-induced cell viability, cell proliferation and chromosome misalignment

**Fig. 5:**
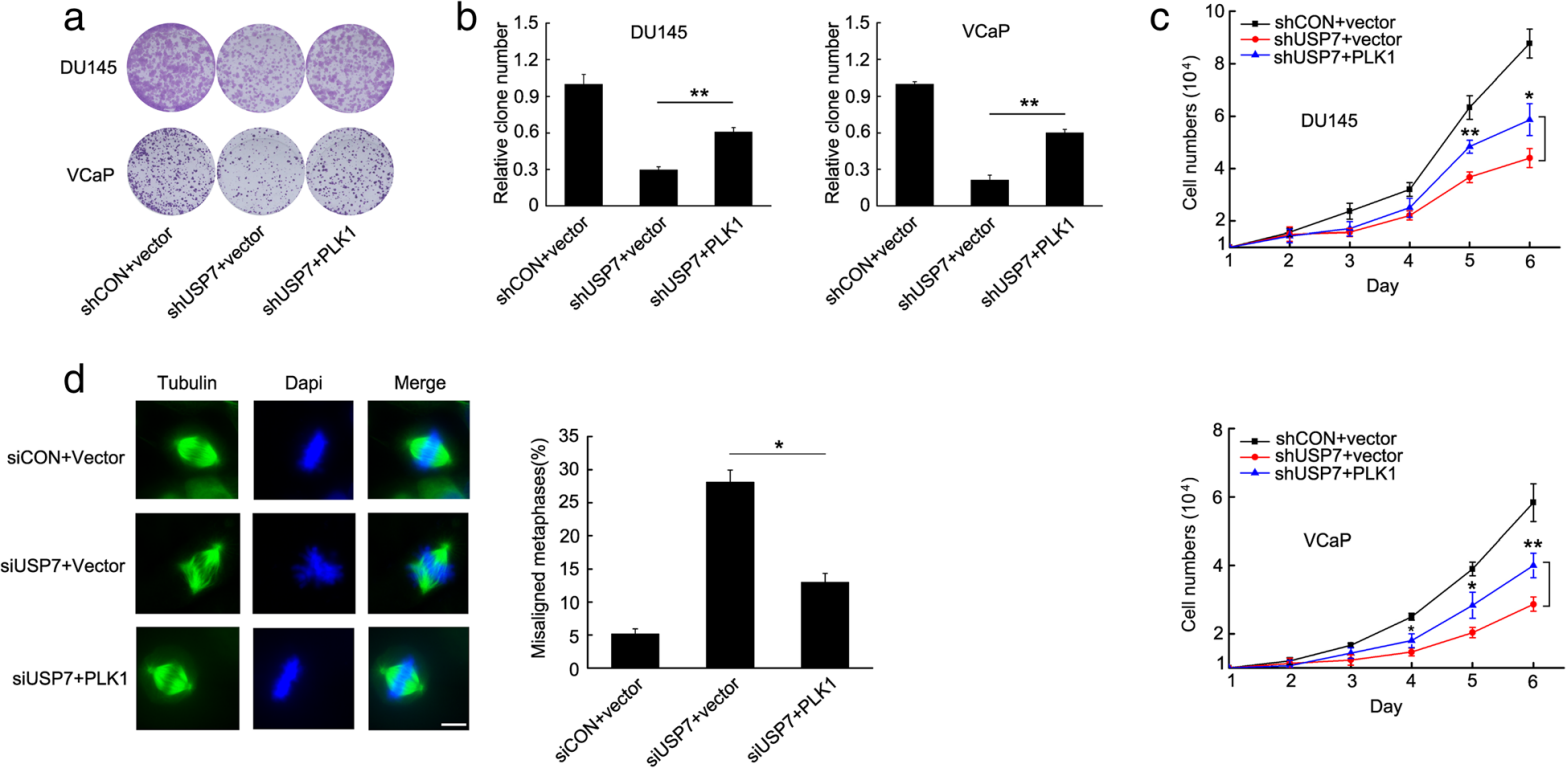
USP7 knockdown retarded cell proliferation and induced G2/M cell cycle arrest and chromosome misalignment by targeting PLK1. DU145 and VCaP cells stably expressing control or USP7 shRNAs were transiently transfected with PLK1 plasmid. **a** and **b** The same number of cells were seeded for colony formation. A representative experiment out of three independent experiments is shown. Relative numbers of cell colonies were quantified. **c** The cells were trypsinized and counted at the indicated time points. **d** DU145 cells were transiently co-transfected with control or USP7 siRNAs together with PLK1 plasmid. Spindles were stained with an immunofluorescent α-tubulin antibody, and nuclear DNA were stained with DAPI. Scale bar, 10 μM. The number of cells with chromosome misalignment were quantified

To test USP7’s biological functions by regulating PLK1, we artificially elevated PLK1 when USP7 was knocked down by RNAi (Additional file 3: Figure S3A) and tested cell colony formation, cell proliferation and chromosome status. As expected, USP7 knockdown with shRNA significantly decreased cell colony formation and retarded cell proliferation, but PLK1 elevation rescued cell colony formation and cell proliferation (Fig. 5a, b, c). Furthermore, USP7 knockdown induced chromosome misalignment in mitosis, but artificial elevation of PLK1 restored chromosome alignment in mitosis (Fig. 5d). These results validated the critical role of USP7 in cell proliferation and cell cycle transition by regulating PLK1.

### USP7 is positively correlated with PLK1, and contributes to taxane resistance

**Fig. 6:**
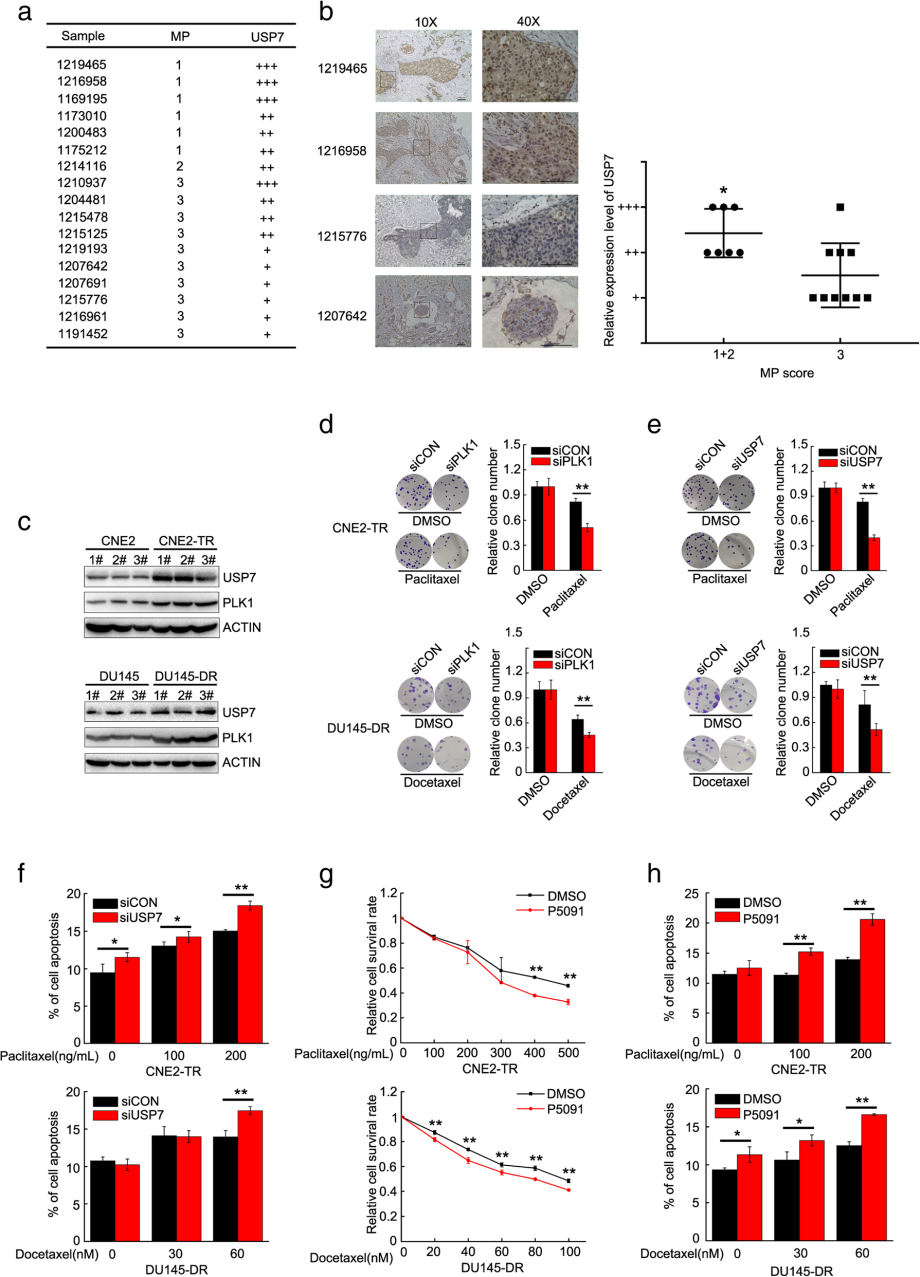
USP7 is positively correlated with PLK1, and contributes to taxane resistance. **a** Expression levels and localizations of USP7 protein were detected by IHC in breast cancer tissues. **b** Summary of USP7 IHC positive signal and MP (Miller-Payne) score in primary breast tumor sections. USP7 expression levels were negatively correlated with MP score by statistical analysis using the Mann-Whitney test. **c** Paclitaxel-resistant CNE2-TR or docetaxel-resistant DU145-DR cells were generated, and USP7 and PLK1 proteins were assessed by western blot. **d** and **e** PLK1 or USP7 were depleted with siRNA in CNE2-TR and DU145-DR cells, and the cells were treated with DMSO, paclitaxel (100 ng/mL) or docetaxel (40 nM) for 48 h. The same number of cells were seeded for colony formation. Relative numbers of cell colonies were quantified. **f** USP7 was depleted with siRNA in CNE2-TR or DU145-DR cells, and cells were treated with paclitaxel or docetaxel for 24 h. The cells were stained with Annex-V/PI, and apoptotic cells were detected by flow cytometry. **g** CNE2-TR or DU145-DR cells were treated with or without P5091 (2 μM) together with paclitaxel or docetaxel at the doses shown for 24 h. Cell viability was tested by MTS assay. **h** CNE2-TR or DU145-DR cells were treated with or without P5091 (2 μM) together with paclitaxel or docetaxel at the doses shown for 24 h. The cells were stained with Annex-V/PI, and apoptotic cells were detected by flow cytometry

We tested the expression and intercellular localization of USP7 protein by immunohistochemical staining in tissue sections of primary breast cancer. The sensitivity of tumor tissues to taxane was evaluated by the MP (Miller-Payne) grading scale, a system for the evaluation of patient response to taxane chemotherapy. The expression of USP7 is very high in breast cancer sections with low MP scores, while USP7 levels are low in MP high breast cancer sections (Fig. 6a). Thus USP7 expression levels were negatively correlated with MP scores (Fig. 6b). The data further validated that USP7 may be a reliable biomarker of taxane resistance, in addition to a biomarker of cancer malignancy.

Paclitaxel or docetaxel is the first-line chemotherapeutic drug for prostate cancer and nasopharyngeal carcinoma patients. However, taxane resistance often develops after a period of treatment. We developed two taxane-resistant cell line, CNE2-TR and DU145-DR. The resistance of cells to taxane were assessed by cell viability assay (Additional file 2: Figure S2A-B) and apoptosis detectionassay (Additional file 2: Figure S2C-D). We found higher expression of both USP7 and PLK1 proteins in docetaxel-resistant DU145-DR cells and paclitaxel-resistant CNE2-TR cells (Fig. 6c), and PLK ubiquitinated protein levels were decreased in taxane-resistant cells (Additional file 1: Figure S1C). The knockdown of USP7 or PLK1 in DU145-DR or CNE2-TR cells significantly weakened colony-formation ability (Fig. 6d, e). The knockdown efficiency of USP7 and PLK1 were tested by western blot (Additional file 3: Figure S3B-C). USP7 depletion also significantly increased the population of apoptotic cells in the two cell lines (Fig. 6f). Consistently, the USP7 inhibitor P5091 significantly retarded cell proliferation and increased the population of apoptotic cells in both DU145-DR and CNE2-TR cells (Fig. 6g, h).

## Discussion

USP7 has been identified as an oncogene with an important role in carcinogenesis and therapeutic resistance in a variety of cancer types. USP7 has also been regarded as an effective anticancer target, and several small molecular inhibitors have been developed to target USP7. However, no DUB inhibitor has stepped up to clinical trials, though the USP7 inhibitor P5091 showed significant anti-cancer effects in pre-clinical tests. In our present study, USP7 inhibition by P5091 significantly retarded cell proliferation and reduced cell colony formation, and USP7 inhibition by P5091 induced G2/M cell cycle arrest and cell apoptosis.

The anti-cancer effect of USP7 knockdown is associated with interference in some cancer-associated pathways, such as p53-MDM2 axis, Ki-67, c-Myc, FOXO, PTEN and Claspin, which are critical for DNA damage repair, epigenetic regulation and immune responses [18, 32]. Unexpectedly, we for the first time found that USP7 knockdown induced chromosome misalignment in mitosis. The exact molecular mechanism by which USP7 controls mitosis is currently unknown.

By using high-throughput proteomics methods, we detected USP7 protein in the protein complex immunoprecipitated by PLK1 antibody, and the interaction of USP7 and PLK1 was validated by co-immunoprecipitation. The PBD domain of PLK1, especially two polo motifs in the domain, interacts with USP7 protein. USP7 sustained PLK1 protein stability, and conversely USP7 inhibition by P5091 promoted PLK1 protein degradation through the ubiquitination-proteasome pathway. It is well known that PLK1 is a master mitotic regulator, and controls centrosome maturation, entry into mitosis, chromosome segregation and cytokinesis [33, 34]. PLK1 inhibition results in aberrant mitotic progression. PLK1 inhibition prevents the formation of a bipolar spindle and misalignment of chromosomes in the metaphase plate. PLK1 inhibitors have been developed for cancer therapy. Overexpressing PLK1 reversed USP7 knockdown by RNAi and restored chromosome alignment in mitosis. These data validated that USP7 controls mitosis by regulating PLK1.

USP7 and PLK1 have strong clinical relevance, being overexpressed in tumor cells in a many cancer types. The degree of intratumoral overexpression closely correlates with poor patient prognosis and sensitivity to taxane chemotherapy (MP). Both USP7 and PLK1 have been considered bona fide cancer targets, and USP7 or PLK1 inhibitors have been developed to experimentally treat different cancer types or to re-sensitize cancer cells to overcome therapeutic resistance.

## Conclusion

In this study we report for the first time that USP7 knockdown resulted in aberrant mitosis, and the effect was achieved via PLK1, a critical mitotic regulator. Furthermore, we report for the first time that PLK1 is a novel substrate of USP7 deubiquitinase, and that USP7 sustained the protein stability of PLK1. USP7 knockdown or inhibition induced cell apoptosis and cell cycle G2/M arrest and overcame taxane resistance by inducing the protein degradation of PLK1, resulting in chromosome misalignment in mitosis. The single or combined administration of USP7 or PLK1 inhibitors has demonstrated great anticancer potential for future clinical use.

## Supplementary information

**Additional file 1. Figure S1.** (A-B) DU145 and VCaP were transfected with a scramble or two different USP7 siRNAs for 48 h.

**Additional file 2. Figure S2.** Assessment of drug resistance of paclitaxel-resistant and docetaxel-resistant cells.

**Additional file 3. Figure S3.** USP7 and PLK1 protein levels were evaluated by western blotting.

**Additional file 4. Figure S4.** The original blots.

## Data Availability

The data that support the findings of this study are available from the corresponding authors upon reasonable request.

## Abbreviations

CHX: Cycloheximide
CST: Cell Signaling Technology
DUB: Deubiquitinating enzymes
HAUSP: Herpes-associated ubiquitin-specific protease
HSV: Herpes simplex virus
MCE: MedChemExpress
MP: Miller-Payne
PBD: Polo-box domains
PLK1: Polo-like kinase 1
SD: Standard Deviation
UPS: Ubiquitin proteasome system
USP7: Ubiquitin specific protease 7
USPS: Ubiquitin-specific proteases
