# Supplementary figures and images for: USP7 is a novel Deubiquitinase sustaining PLK1 protein stability and regulating chromosome alignment in mitosis
Source: J Exp Clin Cancer Res. 2019 Nov 15;38:468. doi: 10.1186/s13046-019-1457-8 (PMC6858727; doi:10.1186/s13046-019-1457-8)

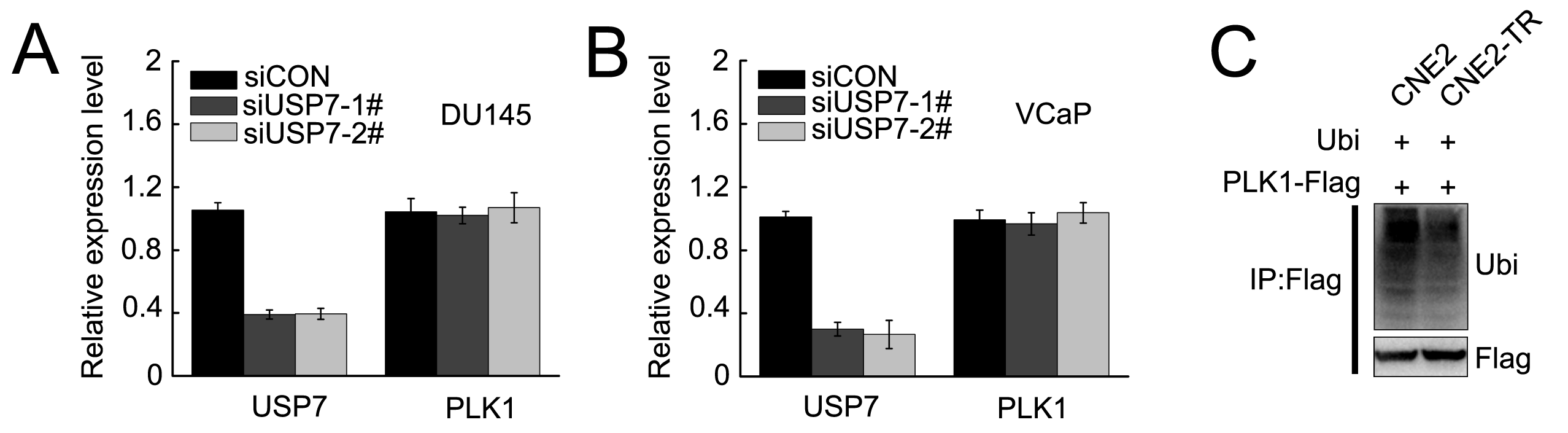

Supplement: Supplementary file 1 — Additional file 1. Figure S1. (A-B) DU145 and VCaP were transfected with a scramble or two different USP7 siRNAs for 48 h. [file 13046_2019_1457_MOESM1_ESM.tif]

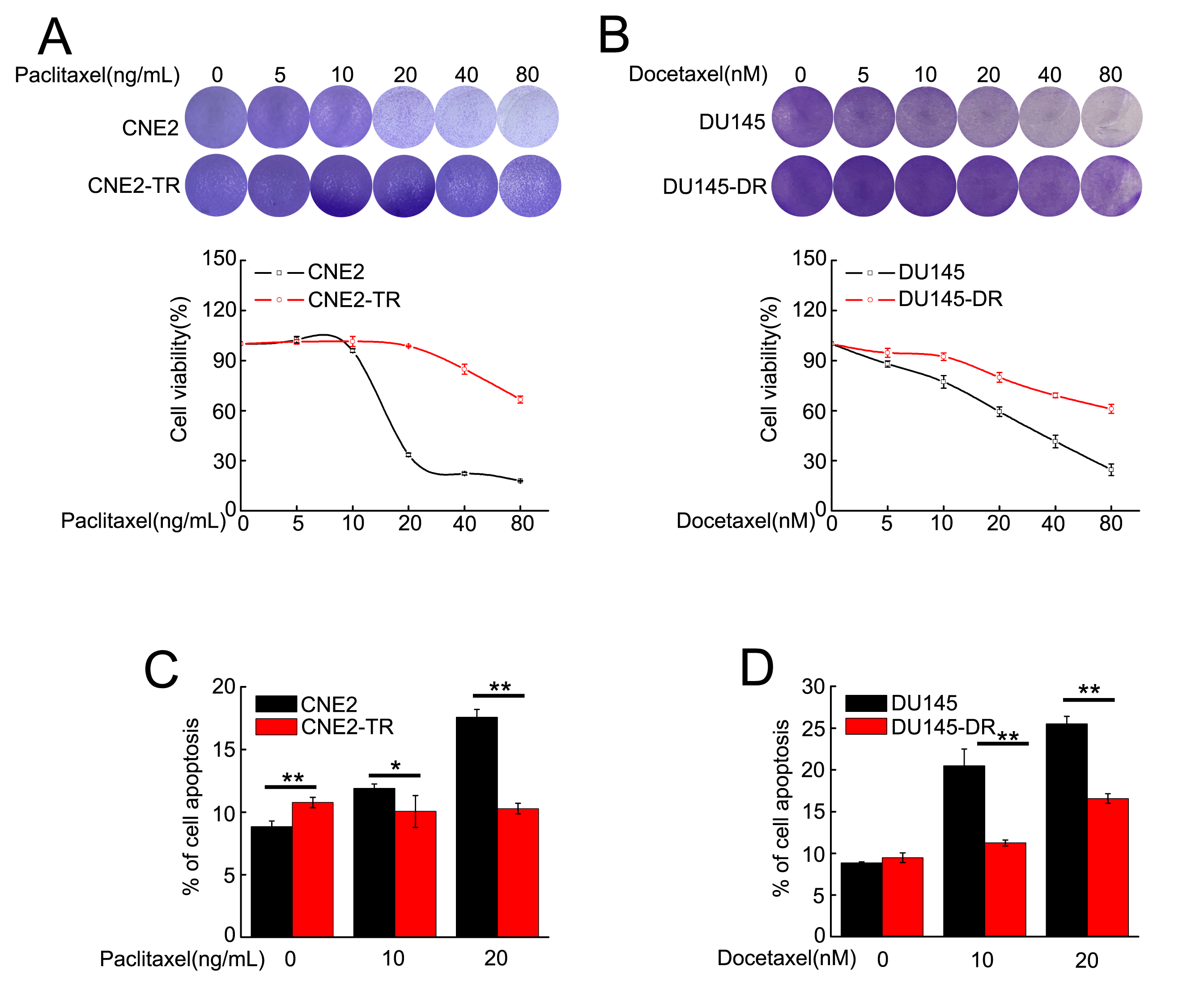

Supplement: Supplementary file 2 — Additional file 2. Figure S2. Assessment of drug resistance of paclitaxel-resistant and docetaxel-resistant cells. [file 13046_2019_1457_MOESM2_ESM.tif]

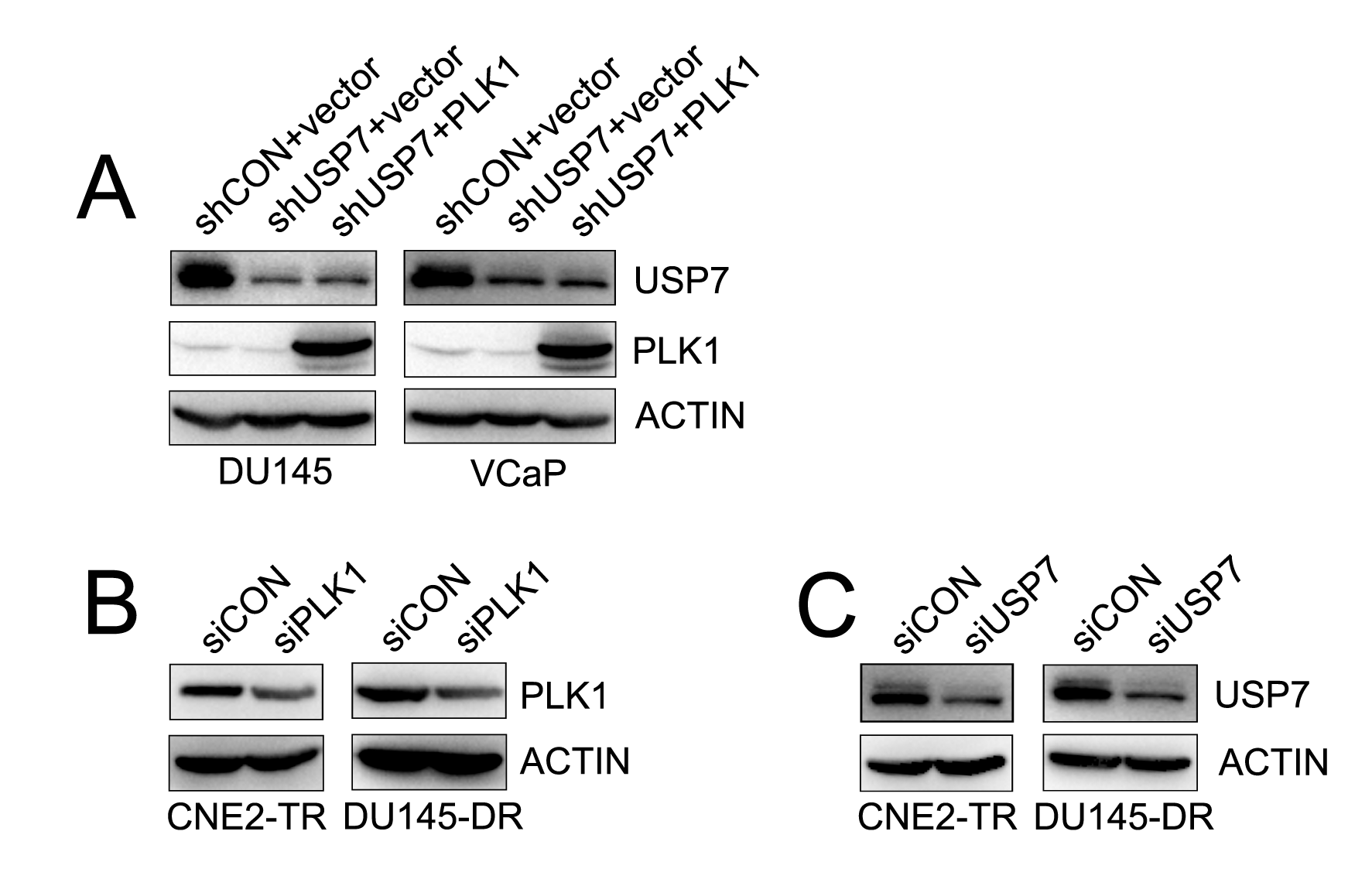

Supplement: Supplementary file 3 — Additional file 3. Figure S3. USP7 and PLK1 protein levels were evaluated by western blotting. [file 13046_2019_1457_MOESM3_ESM.tif]

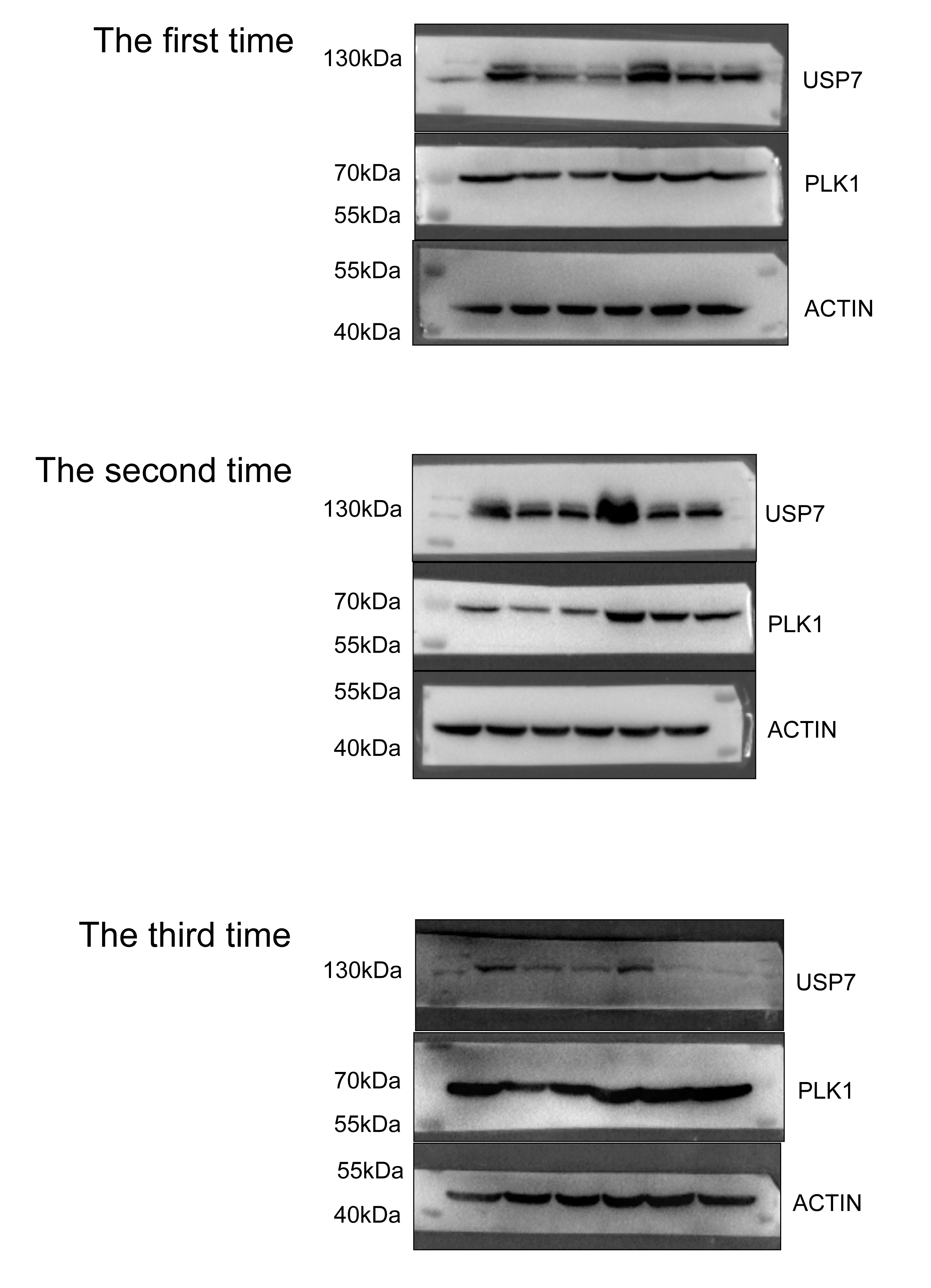

Supplement: Supplementary file 4 — Additional file 4. Figure S4. The original blots. [file 13046_2019_1457_MOESM4_ESM.tif]
